# Characterization of Chlorhexidine Resistance Among Clinical Isolates of Coagulase-Negative Staphylococcus Species in a Tertiary Care Center, Chennai

**DOI:** 10.7759/cureus.71041

**Published:** 2024-10-07

**Authors:** Sanjana Paranji Srirama, Shanthi Mariappan, Uma Sekar, Rhea Michelle J Khodabux

**Affiliations:** 1 Microbiology, Sri Ramachandra Institute of Higher Education and Research, Chennai, IND

**Keywords:** antiseptic resistance in cons, chlorhexidine resistance in cons, hospital infection control, qaca/b in cons, smr in cons

## Abstract

Background

Coagulase-negative *Staphylococci *(CoNS) are potential pathogens and are often associated with healthcare-associated infections (HAIs). Chlorhexidine (CHX) is the most widely used antiseptic to reduce colonization and infection by all *Staphylococci*, including CoNS. Resistance to CHX among CoNS has been observed over the past few years, consequent to its widespread use. Phenotypic tolerance or reduced susceptibility to CHX is conferred by plasmid-mediated *qac* group of genes, mainly *qacA/B* and *smr*, which cause activation of efflux pumps over the bacterial cell wall. This study aims to characterize the phenotypic and genotypic resistance exhibited by CoNS species against CHX.

Methods

After ethical approval, 148 consecutive, non-repetitive isolates of clinically significant CoNS species of hospitalized patients, isolated from blood samples and exudative specimens, were included in the study. Speciation was performed by conventional biochemical identification and automated methods. Antimicrobial susceptibility testing was performed by disc diffusion technique and for vancomycin by minimum inhibitory concentration (MIC) determination, as per Clinical Laboratory Standards Institute (CLSI) M-100 2023 guidelines. Methicillin resistance was detected using a cefoxitin disc. MIC for CHX was performed by agar dilution method; reduced susceptibility was considered when MIC to CHX ≥4 µg/mL. The simplex polymerase chain reaction (PCR) was carried out with suitable controls to detect *qacA/B* and *smr*. Statistical analysis was conducted to determine the association of *qacA/B* and *smr* genes with MIC of CHX in the study isolates.

Results

Fifteen different species of CoNS were obtained from clinical samples. A high percentage of resistance was observed against various classes of antibiotics. Methicillin resistance was observed in 69.6% (103/148) of isolates. Of 148 CoNS, 52.7% (78/148) of isolates exhibited reduced susceptibility to CHX with an MIC ≥4 µg/mL. These isolates exhibited a higher percentage of methicillin resistance (75.6%, 59/78). By PCR, 34.5% (51/148) of isolates carried either or both genes. Gene *qacA/B* was solely detected in 27.02% (40/148) of isolates, of which 14 were CHX-tolerant and the remaining 26 were CHX-susceptible. Gene *smr* was solely detected in 4.1% (6/148) of isolates comprising three isolates each in CHX-tolerant and susceptible categories*. *There were 3.4% (5/148) of isolates that harbored both genes, of which only one isolate was CHX-susceptible, while the other four were CHX-tolerant. A proportion of isolates that were phenotypically tolerant to CHX did not carry either or both genes. A significant statistical association was found between reduced susceptibility to CHX and the presence of antiseptic resistance genes in the study isolates (p-value=0.033942).

Conclusion

To our knowledge, this is the first study from South India to investigate CHX resistance among CoNS using phenotypic and genotypic methods. The rise of antiseptic resistance among CoNS is an emerging threat to current infection control practices. The presence of *qacA/B *and* smr* genes, especially in CHX susceptible isolates, is concerning since these resistance genes are located on transferable plasmids, and the isolates can develop resistance eventually upon exposure to CHX.

## Introduction

Coagulase-negative *Staphylococci *(CoNS) are a part of the normal flora of human skin and mucous membranes and have been described as skin commensals and non-pathogenic. However, extensive research over the past two to three decades has revealed that CoNS are a significant cause of healthcare-associated infections (HAIs) and are clinically relevant pathogens, particularly in neonates and critically ill patients, those requiring prolonged hospitalization, individuals with indwelling medical devices, and those with compromised immune function [1,2]. More recent reports indicate that approximately 11% to 35% of bloodstream infections (BSIs) in intensive care patients and neonates are caused by various species of CoNS, more commonly by *S. haemolyticus, S. hominis, S. epidermidis, *and *S. saprophyticus* [3-5].

Colonization of various anatomical sites is the primary source of endogenous infections by CoNS, though up to 40% of infections can result from transmission by healthcare personnel [1]. These infections are becoming increasingly difficult to treat due to the development of antimicrobial resistance and virulence associated with biofilm formation. Most infection prevention and control measures are focused on maintaining the asepsis of healthcare providers and the hospital environment [2]. The application of several antiseptics, with chlorhexidine (CHX) being the most common agent, has been practiced as an infection control tool to curtail HAIs.

CHX is a reliable biguanide antiseptic that is available in various forms, such as hand rub (0.5-4%), body wash (2-4%), and oral rinse (0.12-0.2%). It is used for topical antisepsis and the mechanical disinfection of surfaces and devices in community and hospital settings. CHX has excellent antimicrobial activity against gram-positive bacteria, as it enters the cell membrane of the microbe, causing cytolysis, release of intracellular components, and coagulation and precipitation of the cytoplasmic proteins leading to cell death [6]. The lipophilic and positively charged properties of CHX contribute to the destruction of gram-positive cocci, leading to bacterial cell death at higher concentrations. Although there are several compounds available for use as antiseptics and disinfectants, the literature states that CHX has a safer and superior spectrum of action than other biocides [7].

The development of resistance to CHX in *Staphylococci*, especially CoNS, has been on the rise. This is due to the acquisition of a major facilitator superfamily (MFS) and small multidrug resistance group (SMR), which are determinants in the plasmid-mediated *qac* group of genes, mainly *qacA/B* and *smr*. The major resistance mechanisms exhibited against CHX are by activation of efflux pumps in the bacterial cell wall, change in membrane permeability, and extensive formation of biofilm, which hinders intracellular penetration [7].

Formulation of a proper definition for CHX resistance has proven to be challenging. Traditionally, an organism is deemed as susceptible if the concentration of the employed antibiotic or antiseptic agent is efficient enough to neutralize it [7,8]. Unlike antibiotics, antiseptics do not have a specific minimum inhibitory concentration (MIC) or minimum bactericidal concentration (MBC) breakpoints or guidelines to categorize an organism as susceptible or resistant. Tests to determine antiseptic efficacy are also not performed routinely in laboratories. Hence, the term "antiseptic/biocide resistance" is often vague and indiscriminate. To overcome these shortcomings, better terminologies such as "antiseptic tolerance" or "reduced susceptibility" have been employed in order to evaluate the efficiency of antiseptic agents [8]. An important factor that may be responsible for the development of tolerance or reduced susceptibility is the selective environmental pressure by the intensive use of CHX, which may also drive the overexpression of genes conferring resistance. In any case, the presence of efflux-mediated resistance genes can be described as genotypic resistance [7,8]. Genotypic methods are valuable for the identification or confirmation of sequences known to encode antiseptic resistance genes.

US Centers for Disease Control and Prevention (CDC) states that at least one in 31 patients in a hospital suffers from at least one HAI (https://www.cdc.gov/healthcare-associated-infections/php/data/index.html). Approximately one to 1.5 million surgical site infections are being reported annually [1]. Several healthcare facilities around the world advocate the use of body washes containing CHX for intensive care patients as a measure to reduce colonization by commensals. Additionally, daily treatment or bathing of patients with CHX soap and skin antisepsis prior to surgery and invasive procedures are essential to prevent HAIs [1]. The presence of antiseptic resistance mechanisms and genes in clinical isolates of CoNS is a cause for concern because it threatens current infection control practices. The objective of the present study was primarily to assess the prevalence of tolerance and genotypic resistance to CHX among various species of pathogenic CoNS isolated from hospitalized patients at a tertiary care center.

## Materials and methods

Collection and identification of isolates

The study was undertaken at a 1600-bed teaching hospital in Chennai, South India, for a period of one year from August 2022 to July 2023. The study protocol was approved by the institutional ethics committee with a waiver of informed consent (CSP-MED/22/AUG/78/101). A total of 148 consecutive, non-repetitive isolates of clinically significant CoNS* *were included in this study. The sources of these isolates were blood (n=58) and exudative specimens (n=90), such as pus, tissue, and exudative fluid from sterile body sites. The significance of the isolates was based on correlation with clinical diagnosis, the presence of intracellular organisms in gram stain, and significant growth on culture media.

Species-level identification was done by conventional biochemical tests and automated methods (matrix-assisted laser desorption ionization-time of flight mass spectrometry (MALDI-ToF MS): bioMérieux, Marcy l’Etoile, France). Conventional speciation of CoNS was performed using slide and tube coagulase tests, ornithine decarboxylation test, Voges-Proskauer (VP) test, nitrate reduction test, urease test, susceptibility to novobiocin (5 μg) and polymyxin B (300 units) discs, and fermentation of sugars such as mannose, mannitol, trehalose, lactose, sucrose, and xylose [9].

Antimicrobial susceptibility testing

Antimicrobial susceptibility testing was performed by disc diffusion method in accordance with the Clinical Laboratory Standards Institute (CLSI) 2023 guidelines for ampicillin (10 µg), erythromycin (15 µg), clindamycin (2 µg), gentamycin (30 µg), ciprofloxacin (5 µg), and linezolid (30 µg) and for vancomycin by MIC method [10]. Methicillin resistance was determined by using a cefoxitin (30 µg) disc (Himedia Laboratories, Mumbai, Maharashtra, India).

Minimum inhibitory concentration for chlorhexidine

MIC for CHX digluconate solution (C9394; Sigma) was determined by the agar dilution method. The plates were prepared from a stock solution of CHX digluconate containing 160 µg/mL. The plates were prepared in six different concentrations ranging from 0.5 to 16 µg/mL [11,12]. Three to four colonies of the collected isolates were inoculated into peptone broth adjusted to a 0.5 McFarland standard for 20 minutes and were then inoculated onto gridded Mueller-Hinton agar plates using a sterile loop in the designated grids. The plates were incubated at 37°C, and readings were taken after 24 hours. Isolates were considered to be phenotypically tolerant to CHX if the MIC was ≥4 µg/mL considering previously published reports [8,13].

Detection of antiseptic resistance genes

The screening of clinical isolates for potential genetic markers of antiseptic resistance is highly beneficial due to the lack of standardized testing methods and susceptibility breakpoints indicative of phenotypic tolerance or reduced susceptibility to these agents [6,7]. Simplex polymerase chain reaction (PCR) was carried out on all collected isolates to detect the presence of the genes encoding for resistance to CHX. The genes investigated were *qacA/B* and *smr*.

The template DNA was extracted by the boiling lysis method [14]. The PCR mixture was prepared for a reaction volume of 10 µL and contained 5 µL of master mix, 2 µL of sterile nuclease-free water, 0.5 µL of forward primer, 0.5 µL of reverse primer, and 2 µL of extracted DNA. The details of the resistance genes with their respective forward and reverse primers are given in Table 1 [11,15].

**Table 1:** Primer sequences for the tested genes bp, base pairs

Primers	Primer sequence (5′-3′)	Product size
*qacA/B *forward	GCA GAA AGT GCA GAG TTC G	361 bp
*qacA/B *reverse	CCA GTC CAA TCA TGC CTG	
*smr *forward	GCC ATA AGT ACT GAA GTT ATT GGA	195 bp
*smr *reverse	GAC TAC GGT TGT TAA GAC TAA ACC T	

PCR cycling conditions for both genes were denaturation at 96°C for three minutes, 25 cycles at 95°C for 20 seconds, 53°C for 20 seconds, and 72°C for 20 seconds, and final elongation at 72°C for five minutes [15]. The amplicons were separated in a 2% agarose gel containing ethidium bromide. The gels were run in 1X tris-acetate-EDTA (TAE) buffer at 100V for 25 minutes. The gels were then visualized using the ultraviolet transilluminator.

PCR-positive amplicons, that were detected for each gene, were purified and sequenced. The sequenced strains for each gene were used as positive controls. Sequencing was done by Sanger ABI 3130xl DNA analyzing instrument (Biokart, Bangalore). Using the Bio edit sequence program (product version 7.0.5.3), the nucleotide sequences were aligned and were compared with the basic alignment search tool offered on the National Centre for Biotechnology Information website (www.ncbi.nlm.nih.gov). The nucleotide sequences were analyzed and then submitted to GenBank and the accession numbers were obtained (*qacA/B *- OQ835625; *smr *- OQ835626).

Statistical analysis

Statistical analysis was performed using SPSS software 17.0 version (IBM Inc., Armonk, New York). A chi-square test was conducted to compare the distribution of MIC values to CHX and antiseptic resistance genes among all species of CoNS. A chi-square test was also conducted to compare the presence of antiseptic resistance genes with methicillin resistance in CoNS. A p-value of less than 0.05 was considered statistically significant.

## Results

Speciation and antimicrobial susceptibility testing

CoNS isolates were collected from 90 exudative specimens and 58 blood samples (n=148). Fifteen different species of CoNS were identified and the most commonly isolated species was* S. haemolyticus.*

The percentage of resistance exhibited by CoNS against various classes of antibiotics was as follows: ampicillin - 89.2% (132/148), erythromycin - 78.4% (116/148), clindamycin - 38.9% (59/148), gentamycin - 18.9% (28/148), ciprofloxacin - 44.4% (66/148), and linezolid - 2.02% (3/148). Of the 148 CoNS isolates, 69.6% (103/148) were resistant to cefoxitin. The three linezolid-resistant isolates were identified as *S. cohnii ssp. urealyticum* (n=2) and *S. haemolyticus* (n=1). All CoNS isolates were susceptible to vancomycin.

Minimum inhibitory concentration method for chlorhexidine digluconate

Of the 148 identified CoNS, 52.7% (78/148) of isolates exhibited reduced susceptibility to CHX with MIC ≥4 µg/mL. The remaining 47.3% (70/148) of isolates were susceptible to CHX. Among CHX-tolerant isolates, 42.3% (33/78) belonged to *S. haemolyticus*, whereas among CHX-susceptible isolates, 40% (28/70) belonged to *S. hominis*. The CHX-tolerant isolates also showed a higher percentage of methicillin resistance (75.6%, 59/78). The range of MIC for CHX among CoNS species is displayed in Table 2.

**Table 2:** Distribution of CoNS isolates according to MIC value to CHX CoNS, coagulase-negative *Staphylococci*; MIC, minimum inhibitory concentration; CHX, chlorhexidine

Identified species	n	%	MIC <4 µg/mL	MIC ≥4 µg/mL
S. haemolyticus	51	34.5	18	33
S. hominis	41	27.7	28	13
S. epidermidis	28	18.9	15	13
S. cohnii ssp. cohnii	4	2.7	1	3
S. lugdunensis	4	2.7	1	3
S. capitis ssp. urealyticus	4	2.7	2	2
S. warneri	3	2.03	1	2
S. saprophyticus	3	2.03	1	2
S. capitis ssp. capitis	2	1.34	0	2
S. caprae	2	1.34	0	2
S. cohnii ssp. urealyticum	2	1.34	0	2
S. arlettae	1	0.68	1	0
S. gallinarum	1	0.68	0	1
S. pasteuri	1	0.68	1	0
S. xylosus	1	0.68	1	0

Genes encoding for chlorhexidine resistance

**Figure 1:**
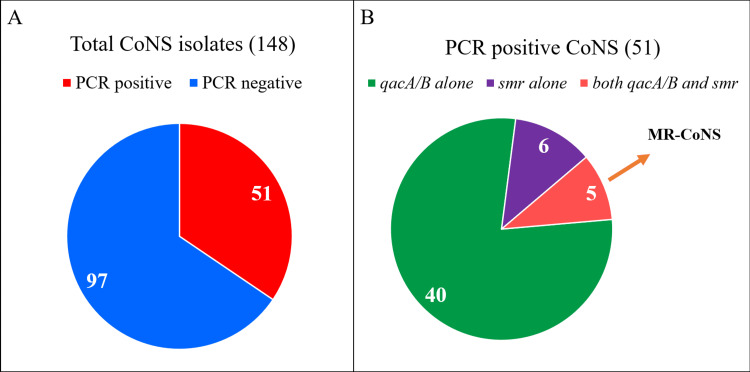
Representation of PCR-positive isolates among CoNS A) PCR positive and negative isolates among all 148 study isolates of CoNS. B) PCR-positive isolates based on the genes identified among 51 PCR-positive isolates. CoNS, coagulase-negative *Staphylococci*; PCR, polymerase chain reaction

**Figure 2:**
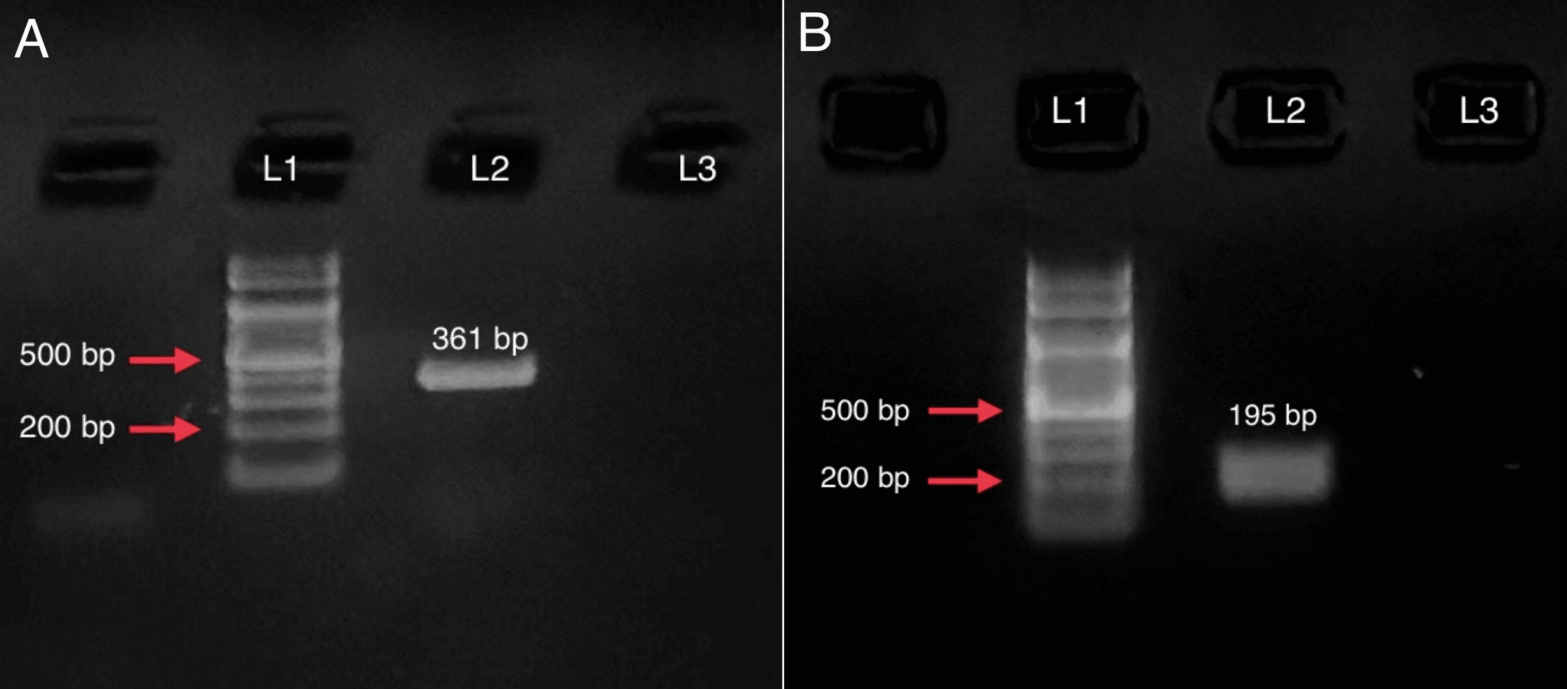
Gel electrophoresis picture of the sequenced PCR amplicons for the tested genes A) Lane 1-100 bp DNA ladder, lane 2-gene *qacA/B* (361 bp), and lane 3-negative control. B) Lane 1-100 bp DNA ladder, lane 2-gene *smr *(195 base pairs), lane 3-negative control. bp, base pairs; CoNS, coagulase-negative *Staphylococci; *PCR, polymerase chain reaction

Of all 148 CoNS isolates, 34.5% (51/148) carried either or both genes (Table 3 and Figure 1). Gene *qacA/B* (Figure 2A) was exclusively detected in 27.02% (40/148) of isolates, of which 14 were CHX-tolerant and the remaining 26 were CHX-susceptible. Gene *smr *(Figure 2B)* *was solely detected in 4.1% (6/51) of isolates, thus comprising three isolates each in the CHX-tolerant and susceptible categories. The presence of both genes was detected in 3.4% (5/51) of isolates, of which only one isolate was CHX-susceptible, while the other four were CHX-tolerant. A high percentage of methicillin resistance was observed among the PCR-positive isolates (80.4%, 41/51).

**Table 3:** Distribution of CoNS study isolates carrying the antiseptic resistance mediating genes CoNS, coagulase-negative *Staphylococci*; PCR, polymerase chain reaction

Identified species	Number of isolates (n)	PCR-positive isolates		
		*qacA/B* only	*smr *only	Both *qacA/B *and *smr*
S. haemolyticus	51	16	5	2
S. hominis	41	14	0	1
S. epidermidis	28	7	0	0
S. cohnii ssp. cohnii	4	1	0	0
S. lugdunensis	4	0	0	0
S. capitis ssp. urealyticus	4	0	1	1
S. warneri	3	1	0	0
S. saprophyticus	3	0	0	1
S. capitis ssp. capitis	2	1	0	0
S. caprae	2	0	0	0
S. cohnii ssp. urealyticum	2	0	0	0
S. arlettae	1	0	0	0
S. gallinarum	1	0	0	0
S. pasteuri	1	0	0	0
S. xylosus	1	0	0	0
Total	148	40	6	5

**Figure 3:**
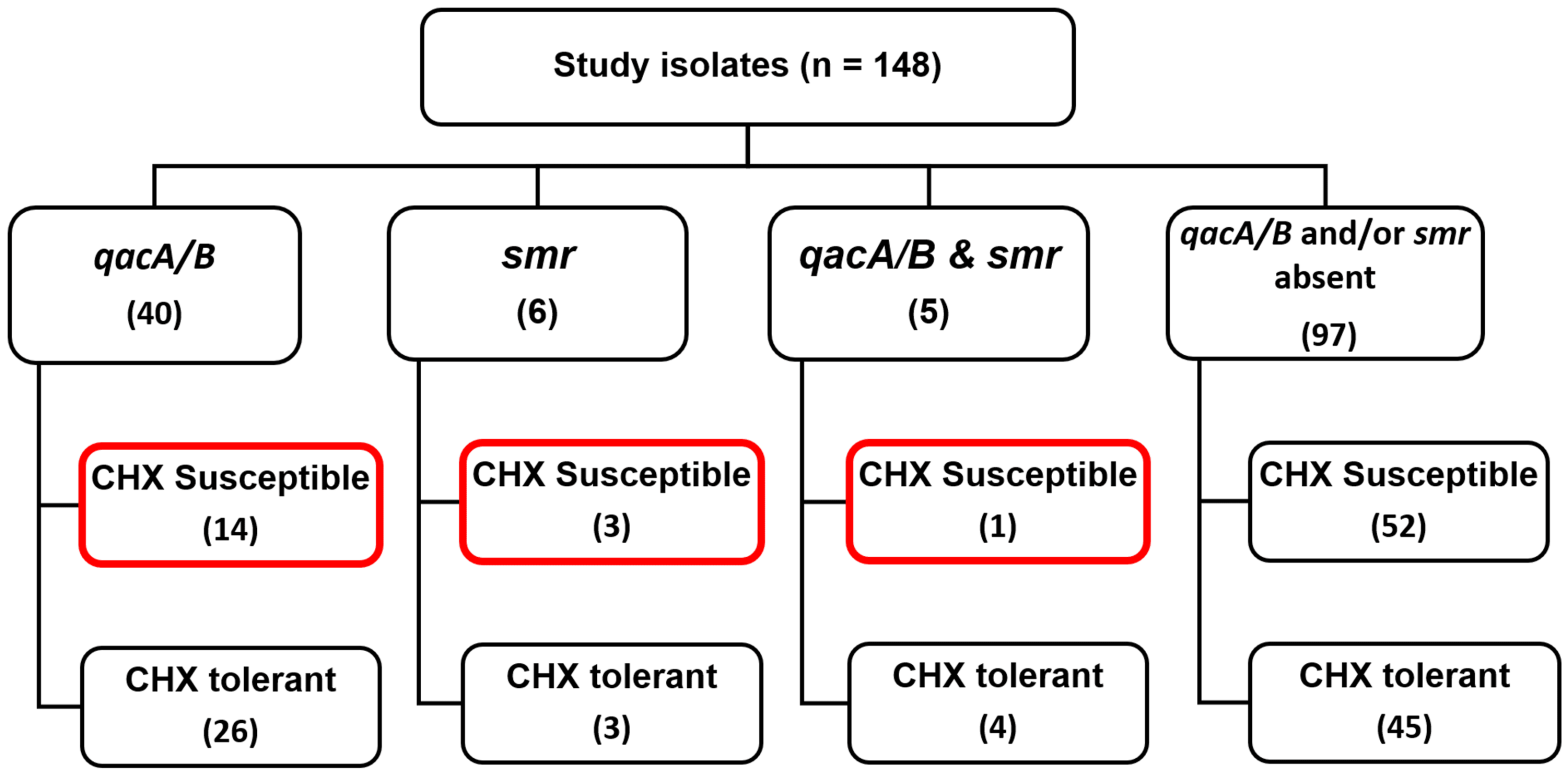
Comparison of phenotypic susceptibility/tolerance versus genotypic resistance in study isolates This figure highlights the PCR-positive isolates with susceptible MIC to CHX (<4 µg/mL). CoNS, coagulase-negative Staphylococci; MIC, minimum inhibitory concentration; CHX, chlorhexidine

Of the 51 PCR-positive isolates (34.5%), 35.3% (18/51) were susceptible to CHX, with MIC ranging from 0.5 to 2 µg/mL (Figure 3). A total of 65.5% (97/148) of isolates did not express either or both *qacA/B *or* smr*. Of these isolates, 46.4% (45/97) were tolerant to CHX by MIC (≥4 µg/mL), and the remaining 53.6% (52/97) were susceptible (Figure 3).

Statistical analysis

Comparison between reduced susceptibility to CHX and the presence of antiseptic resistance genes among all species of CoNS was statistically significant (Table 4). Comparison between the presence of antiseptic resistance genes and methicillin resistance among CoNS was also statistically significant (Table 5).

**Table 4:** Reduced susceptibility to CHX and presence of antiseptic resistance genes among CoNS (p-value <0.05 = statistically significant) *Chi-square test was performed. CoNS, coagulase-negative Staphylococci; CHX, chlorhexidine

CHX resistance encoding genes	CHX tolerant: MIC <4 µg/mL (n=78)	CHX susceptible: MIC ≥4 µg/mL (n=70)	Total (n=148)	p-value*
Present	33	18	51	0.033942
Absent	45	52	97	

**Table 5:** Presence of antiseptic resistance genes vs methicillin resistance among CoNS (p-value <0.05 = statistically significant) *Chi-square test was performed. CoNS, coagulase-negative Staphylococci; CHX, chlorhexidine

CHX resistance encoding genes	Methicillin resistant (n=103)	Methicillin susceptible (n=45)	Total (n=148)	p-value*
Present	41	10	51	0.038398
Absent	62	35	97	

## Discussion

In the present study, 148 clinical isolates of coagulase-negative *Staphylococci *were investigated for susceptibility to antibiotics, phenotypic susceptibility to CHX, and the presence of *qacA/B *and *smr* genes, determinants of resistance to CHX.

In this study, the most isolated CoNS was *S. haemolyticus, *followed by* S. hominis *and *S. epidermidis*. These species have been described as skin commensals with the potential to cause bacteremia and septicemia due to colonization and biofilm formation on intravenous catheters [16]. Several other rare species of CoNS were also identified in this study (Table 2). A few rare but important species, such as *S. lugdunensis, S. urealyticum, S. cohnii, S. saprophyticus, and S. warneri*, have also been implicated in SSTIs and BSIs among all age groups [17]. The clinical significance of CoNS has been gaining attention lately, especially regarding accurate identification, as different species of CoNS can have varying levels of pathogenicity.

CoNS displayed a high percentage of antimicrobial resistance against ampicillin (89.2%), followed by erythromycin (78.4%) in the present study. Methicillin resistance was encountered in 69.6% (103/148) of isolates. Several studies have correlated the association and development of antimicrobial resistance with antiseptic resistance [18-22]. Prompt identification and understanding of drug resistance patterns in CoNS may have an impact on minimizing selection pressure and may help in characterizing potential strains that transfer antibiotic and antiseptic resistance genes to other bacteria.

All 148 isolates were subjected to MIC determination for CHX digluconate by agar dilution method. The isolates were considered resistant to CHX when the MIC was ≥4 μg/mL and the susceptibility breakpoint for CHX applied in this study was derived from the MIC breakpoint from previously published reports [8,12,23]. Phenotypic susceptibility to CHX is mostly based on assays that measure MICs and MBCs. The expected outcome of an interaction between bacteria and an antiseptic agent is lethal action rather than mere inhibition. Measurement of CHX MIC and MBC relates to bacteria tested against much lower concentrations of CHX compared to those achieved in clinical practice [8]. Additionally, the concept of attaining MIC/MBC values in tissues and body fluids for antibiotics is not applicable to antiseptics due to their purpose being limited to topical use. MIC breakpoints for antiseptics are also not as well established as for antibiotics. However, few studies and guidelines suggest that the MIC of CHX ≤2 μg/mL can be used to indicate susceptibility for *Staphylococcus aureus* [8,13]. Others have used epidemiological cut-off values to classify the tested isolates. As CHX can be used in high quantities without causing toxicity, an increase in MIC value does not directly imply "resistance." Tolerance might be a better term for describing increases in MIC instead of using "resistance," which suggests that the disinfectant is no longer effective for its intended use [24]. A high MIC value to CHX can be described as an indicator of poor susceptibility of the organism [19].

With respect to susceptibility to CHX, various studies have cited varying percentages of tolerance by MIC across geographic regions. The prevalence of *Staphylococci *isolates with reduced susceptibility to CHX has been documented at approximately 10-20% in the USA, Australia, and Israel, around 25-30% in Iran and Brazil, and even as high as 70% in Turkey [11-13,20,25]. This difference in percentages could be due to the varying infection control norms and practices across these countries. In this study, 52.7% (78/148) of CoNS displayed reduced susceptibility/tolerance to CHX (MIC ≥4 µg/mL). Among the 78 CHX-tolerant isolates, 42.3% were *S. haemolyticus, *while *S. hominis* constituted 40% among the 70 CHX-susceptible isolates. The occurrence of CHX susceptibility and tolerance was variable among the different species of CoNS in this study. The percentage of methicillin resistance among CHX-tolerant isolates in this study was observed to be higher, similar to a Brazilian study [12]. A report from Israel states that CHX-tolerant *Staphylococci *strains are more often isolated from invasive infections and are likely to be methicillin resistant [20]. Reports state that the possible use of a sub-inhibitory dose of antiseptic and disinfectant solutions, by diluting them, may enhance the MIC value further. Hence, using antiseptic solutions at the concentrations recommended by manufacturers will help prevent some percentage of HAIs. The presence of more than one concomitant resistance determinant also raises the MIC value of CHX [12,26]. In addition, biofilm production by *Staphylococci *may also render these antiseptics ineffective by making them impermeable to biocides [7,20]. However, there is limited data available regarding the phenotypically characterized susceptibility to CHX among CoNS from India.

The lack of standardized breakpoint values for biocide susceptibility testing, combined with other limitations inherent to phenotypic measurement of susceptibility to disinfectants, has hampered the development of standardized assays and, thus led to screening of clinical isolates for potential genetic markers associated with antiseptic resistance. Literature states that the presence of efflux-mediated resistance genes can be described only as genotypic resistance, as the resistance mechanisms are essentially due to overexpression of the antiseptic and biocide resistance genes [6,7]. Genes mediating resistance to CHX act in several ways, such as activating efflux pumps and altering the porin profile of the organism. These genes not only mediate resistance to CHX, but also confer cross-resistance and co-resistance to several routinely used antibiotics [7]. Hence, genotypic studies are beneficial to identify genes mediating biocide and antiseptic resistance.

Fifty-one CoNS isolates (34.5%) carried either or both genes in this study (Table 2 and Figure 1A). Gene *qacA/B* (27.02%, 40/51), rather than *smr*, was carried more commonly by CoNS. A recent study reported that up to 93% of CoNS carry the *qacA/B* gene [18]. A few other studies have also reported that *qacA/B* is more commonly carried than *smr *by *Staphylococcal *isolates [13,18,23,26]. In the current study, *smr *was solely detected in 4.1% (6/51) of isolates and was identified in a relatively lesser proportion of study isolates. Elsewhere, reports have suggested an increased frequency of *smr *than *qacA/B* in *S. aureus* [25]. About 3.4% (5/51) of PCR-positive isolates exhibited the co-existence of both genes, of which 80% (4/5) were CHX-tolerant and all five were methicillin resistant (Figure 1B). An Iranian study states that co-expression of multiple genes was always associated with reduced susceptibility to CHX [18]. Another report states that elevated MIC value, along with the presence of one or more antiseptic resistance mediating genes, can augment the ability of CoNS to persist on the skin despite antisepsis [27]. The statistical correlation between reduced susceptibility to CHX and the presence of antiseptic resistance genes in the study isolates was significant (p-value = 0.033942). The isolates that harbored the genes *qacA/B *and *smr*, either individually or together, fell into both CHX-susceptible and tolerant groups. Hence, it can be inferred that the presence of the gene does not necessitate gene expression as several other factors like transcription regulators, sufficient contact period, and exposure to other antiseptics can also influence the development and expression of tolerance/resistance to CHX. Production of biofilms by CoNS is another significant mechanism contributing to a higher degree of antiseptic failure [26].

The overall PCR-positive isolates exhibited 80.4% (41/51) of methicillin resistance. The statistical correlation between methicillin resistance and the presence of antiseptic resistance genes in CoNS isolates was also significant (p-value = 0.038398). The *Staphylococcal *cassette chromosome, which contains the *mecA* gene, has been reported to integrate plasmids carrying antiseptic resistance genes [7]. Several studies have proposed a possible relationship between the expression of *mecA *and *qacA/B* genes, along with co-resistance to several other routinely used antibiotics [18,21,22,26]. Hence it can be inferred that methicillin resistance not only raises the risk of acquiring antiseptic resistance genes but also poses a threat to the emergence of other antibiotic resistance. These associations, however, were not investigated in this study.

Of the 51 PCR-positive CoNS isolates in this study, 35.3% (18/51) were susceptible to CHX (Figure 3). This suggests either the lack of expression of the genes or their mediation of resistance to other biocides like benzalkonium chloride, tetraphenylphosphonium, desqualinium, and pentaamine, rather than CHX. The antiseptic susceptibility testing of the above-mentioned biocides has not been included in the study protocol.

Of the 97 PCR-negative CoNS isolates, 46.4% (45/97) were phenotypically tolerant to CHX (Figure 3). The presence of other antiseptic resistance mediating genes, namely* qacE, qacF, qacJ, qacG, qacH, norA, LmrS, MdeA, *and *MepA*, encoding the efflux pumps is a probable cause for the elevated MIC to CHX in these isolates. The presence of the above genes needs to be elucidated further. The correlation between the presence of *qacA/B *or *smr* and elevated MIC to CHX is yet to be fully understood. However, some reports suggest that the expression of antiseptic resistance-mediating genes paired with an elevated MIC to CHX may act synergistically, leading to the failure of antisepsis [7,8]. Further studies are hence required not only to understand the mechanisms mediating reduced susceptibility to CHX, but also to investigate *qacA/B *and *smr *mediating resistance to various other biocides. The occurrence of* qacA/B *and *smr* genes among CHX-susceptible isolates is a cause for concern, as these genes are borne on transferable plasmids, and tolerance may be acquired with prolonged exposure.

CHX is an important antiseptic agent used in the prevention of HAIs. It is advisable to routinely monitor the antimicrobial activity of commonly used antiseptics due to the threat of developing resistance mechanisms against these agents. Indiscriminate usage of CHX-containing hand rubs and solutions should be discouraged, and antiseptic stewardship measures should be strengthened to preserve efficacy.

Limitations

Genes such as *qacE, qacF, qacJ, qacG, qacH, norA, LmrS, MdeA, *and *MepA* also mediate CHX tolerance/resistance. The present study isolates were not evaluated for these genes. The presence of a possible association between antibiotic resistance and antiseptic resistance was not investigated in this study.

## Conclusions

In our study, approximately 52.7% of CoNS exhibited reduced susceptibility/tolerance to CHX, and 35.3% of the tested isolates carried either one or both of the genes *qacA/B *and *smr*. With increasing resistance to antibiotics, the development of resistance to antiseptics like CHX poses a threat to the eradication or killing of bacteria in body sites and tissues. Similar to antibiotics, prudent use of antiseptics will help curtail the emergence of tolerance in various gram-positive and gram-negative bacteria. Broader research into the reduced susceptibility of selective organisms to antiseptics and biocides may yield valuable insights that could help prevent HAIs. Recommended concentrations of CHX are used for various purposes based on specific applications, namely 0.5-4% for hand rubs and 2-4% for body washes. Hence, it is mandatory to use apt concentrations of CHX with sufficient contact time for various disinfection practices to reduce selection pressure and curtail tolerance and resistance mechanisms. Strict implementation of infection control practices and antiseptic stewardship measures will mitigate the spread of plasmid-mediated CHX tolerance among bacterial strains. Gene expression studies using real-time PCR with different antiseptics will aid in understanding specific efflux pumps mediating CHX tolerance/resistance. Further studies are also required to demonstrate other genes mediating CHX resistance, apart from *qacA/B *and *smr*.
